# Psychiatric Comorbidity and Length of Stay in a general hospital

**DOI:** 10.1192/j.eurpsy.2023.1230

**Published:** 2023-07-19

**Authors:** R. Fernández Fernández, P. del Sol Calderón, Á. Izquierdo de la Puente, R. Blanco Fernández, M. Martín García

**Affiliations:** 1 Psychiatry, Hospital Universitario Infanta Cristina, Parla; 2 Psychiatry, Hospital Universitario Puerta de Hierro, Majadahonda, Spain

## Abstract

**Introduction:**

Psychiatric comorbidity has a significant impact on the patient’s overall health, with an increased risk of death for those patients with mental-physical comorbidity (Tan et al., 2021). This impacts, among other things, the average hospital stay of a patient with psychiatric comorbidity. For example, an American study shows that psychiatric comorbidity was associated with greater inpatient utilization, including the risk of additional hospitalizations, days of stay, and hospitalization charges (Sayers et al., 2007). Our study aims to confirm these results in patients admitted to a general hospital for any cause and presenting psychiatric comorbidity.

**Objectives:**

To compare the mean length of stay of patients admitted to a general hospital for any cause according to whether they have psychiatric comorbidity or not.

**Methods:**

We made a descriptive retrospective study through the use of electronic medical records. The drug use history and average day of hospitalization were obtained for all patients admitted to the inpatient service of a general hospital during a 3-year period.

**Results:**

The mean length of stay was longer in patients with psychiatric comorbidity (mean = 9.87 days, SD = 15.45) than in patients without psychiatric comorbidity (mean = 5.23 days, SD = 7.16), the difference being statistically significant for the analysis of variance with a small effect size (F = 18.2; p < 0.001, η²=0.038). The assumption of the equality of variances of the two groups is not fulfilled (Levene F = 29.0; p < 0.01) so Welch’s nonparametric test was applied, whose results do not modify those obtained.

**Table:** 

	N	Mean	SD	SE
No psychiatric comorbidity	296	5.23	7.16	0.416
Psychiatric comorbidity	238	9.87	15.45	1.002

**Conclusions:**

Our results are in line with other studies, showing a longer mean length of stay in those patients admitted for any cause and with associated psychiatric comorbidity. This highlights the importance of having an integrated psychiatry service in a general hospital, as Bronson points out, where they find a shorter mean length of stay in units that have integrated, proactive psychiatric care (Bronson et al., 2019).

**References:**

Bronson, B. D., Alam, A., & Schwartz, J. E. (2019). The Impact of Integrated Psychiatric Care on Hospital Medicine Length of Stay: A Pre-Post Intervention Design With a Simultaneous Usual Care Comparison. Psychosomatics.

Sayers, S. L., Hanrahan, N., Kutney, A., Clarke, S. P., Reis, B. F., & Riegel, B. (2007). Psychiatric comorbidity and greater hospitalization risk, longer length of stay, and higher hospitalization costs in older adults with heart failure. Journal of the American Geriatrics Society.

Tan, X. W., Lee, E. S., Toh, M., Lum, A., Seah, D., Leong, K. P., Chan, C., Fung, D., & Tor, P. C. (2021). Comparison of mental-physical comorbidity, risk of death and mortality among patients with mental disorders - A retrospective cohort study. Journal of psychiatric research.

**Disclosure of Interest:**

None Declared

